# Aberrant mechanical loading induces annulus fibrosus cells apoptosis in intervertebral disc degeneration via mechanosensitive ion channel Piezo1

**DOI:** 10.1186/s13075-023-03093-9

**Published:** 2023-07-07

**Authors:** Chenhao Liu, Xiaoxin Gao, Jinhui Lou, Haiyin Li, Yuxuan Chen, Molong Chen, Yuyao Zhang, Zhilei Hu, Xian Chang, Menglin Luo, Yu Zhai, Changqing Li

**Affiliations:** 1 Department of Orthopedics, The Second Affiliated Hospital of Army Medical University (The Third Military Medical University), Chongqing, 400038 China; 2 State Key Laboratory of Trauma, Burns and Combined Injury, Chongqing, 400038 China; 3 Department of Orthopedics, Qinghai Provincial People’s Hospital, Xining, 810007 Qinghai China; 4 Center of Traumatic Orthopedics, People’s Liberation Army 990 Hospital, Xinyang, 464000 Henan China; 5 Department of Orthopedics/Sports Medicine Center, The First Affiliated Hospital of Army Medical University (The Third Military Medical University), Chongqing, 400038 China; 6 Clinical Laboratory, Qinghai Provincial People’s Hospital, Xining, 810007 Qinghai China

**Keywords:** Intervertebral disc degeneration, Piezo1, Annulus fibrosus cells, Aberrant mechanical loading, Apoptosis

## Abstract

**Background:**

Intervertebral disc degeneration (IVDD) is closely associated with the structural damage in the annulus fibrosus (AF). Aberrant mechanical loading is an important inducement of annulus fibrosus cells (AFCs) apoptosis, which contributes to the AF structural damage and aggravates IVDD, but the underlying mechanism is still unclear. This study aims to investigate the mechanism of a mechanosensitive ion channel protein Piezo1 in aberrant mechanical loading-induced AFCs apoptosis and IVDD.

**Methods:**

Rats were subjected to lumbar instability surgery to induce the unbalanced dynamic and static forces to establish the lumbar instability model. MRI and histological staining were used to evaluate the IVDD degree. A cyclic mechanical stretch (CMS)-stimulated AFCs apoptosis model was established by a Flexcell system in vitro. Tunel staining, mitochondrial membrane potential (MMP) detection, and flow cytometry were used to evaluate the apoptosis level. The activation of Piezo1 was detected using western blot and calcium fluorescent probes. Chemical activator Yoda1, chemical inhibitor GSMTx4, and a lentiviral shRNA-Piezo1 system (Lv-Piezo1) were utilized to regulate the function of Piezo1. High-throughput RNA sequencing (RNA-seq) was used to explore the mechanism of Piezo1-induced AFCs apoptosis. The Calpain activity and the activation of Calpain2/Bax/Caspase3 axis were evaluated by the Calpain activity kit and western blot with the siRNA-mediated Calapin1 or Calpain2 knockdown. Intradiscal administration of Lv-Piezo1 was utilized to evaluate the therapeutic effect of Piezo1 silencing in IVDD rats.

**Results:**

Lumbar instability surgery promoted the expression of Piezo1 in AFCs and stimulated IVDD in rats 4 weeks after surgery. CMS elicited distinct apoptosis of AFCs, with enhanced Piezo1 activation. Yoda1 further promoted CMS-induced apoptosis of AFCs, while GSMTx4 and Lv-Piezo1 exhibited opposite effects. RNA-seq showed that knocking down Piezo1 inhibited the calcium signaling pathway. CMS enhanced Calpain activity and elevated the expression of BAX and cleaved-Caspase3. Calpain2, but not Calpain1 knockdown, inhibited the expression of BAX and cleaved-Caspase3 and alleviated AFCs apoptosis. Lv-Piezo1 significantly alleviated the progress of IVDD in rats after lumbar instability surgery.

**Conclusions:**

Aberrant mechanical loading induces AFCs apoptosis to promote IVDD by activating Piezo1 and downstream Calpain2/BAX/Caspase3 pathway. Piezo1 is expected to be a potential therapeutic target in treating IVDD.

**Supplementary Information:**

The online version contains supplementary material available at 10.1186/s13075-023-03093-9.

## Introduction

Lower back pain (LBP) is characterized by high morbidity, high disability rate, and high recurrence rate, which jeopardizes the quality of patients’ life and impose heavy burdens on healthcare expenditure worldwide [1]. IVDD and IVDD-initiated degenerative disc diseases including intervertebral disc herniation, degenerative spondylolisthesis, and spinal stenosis are the common elicitation of LBP [2]. Anatomically, a healthy intervertebral disc (IVD) consists of a gelatinous proteoglycan-rich nucleus pulposus (NP) in the center, a fibrous collagen-rich annulus fibrosus (AF) in the surrounding, and the bilateral cartilaginous endplates (CEP) adjacent to the superior and inferior vertebral bodies. Excessive cellular apoptosis is one of the distinct characteristics during IVDD [3]. In the past few decades, while much of the research progress exploring the underlying mechanisms and novel strategies to ameliorate apoptosis in NP and CEP has been accomplished, much less is achieved in that of AF [4]. Previous studies demonstrate that AF injury can result in IVD structural instability as a mechanism to promote IVDD progression [5–8]. Annulus fibrosus cells (AFCs) apoptosis results in decreased cellularity, which is regarded as a crucial elicitation to AF structural destabilization [9]. Therefore, investigating the contributing factors and underlying mechanism of AFCs apoptosis is of great importance.

Among the contributing factors of cellular apoptosis in IVD, the biomechanical stimulus is a unique factor [8]. IVD functions as a loading absorber and transmitter and provides flexibility to the spine [10]. Aberrant mechanical loading has been proven to be closely associated with apoptosis in NP and CEP [11, 12]. However, the biomechanical characteristic of AF is not similar to NP and CEP. The multilaminar collagen lamellae of AF are strained by intradiscal pressure through two mechanisms: direct radial tensile from the NP expansion and cranial–caudal stretch from the separation of the two endplates [9]. A human AF tissue level analysis exhibited that the tensile hoop stress can reach 12.7 MPa [13]. Several studies have verified the apoptosis of AFCs significantly rises with the elevation of aberrant mechanical loading degree [7, 14–16]. Therapies that inhibit aberrant mechanical loading-induced AFCs apoptosis can result in alleviated IVDD in animal experiments [17, 18]. However, there have not yet been any studies investigating the underlying mechanism that aberrant mechanical loading stimulates AFCs apoptosis.

The Piezo proteins are newly discovered mechanically sensitive ion channel proteins, whose encoding genes (Piezo1 and Piezo2) were identified in 2010 [19]. Piezo participates in the cellular response to external mechanical stimuli, including compression, stretch, gravity, fluid shear, and so on [20, 21]. Piezo helps cells in converting mechanical signals into biological signals by enhancing calcium ions (Ca^2+^) influx and altering subsequent intracellular calcium signaling [22] and then regulating a series of cellular biological processes, including cell proliferation, cell differentiation, cell migration, and cell apoptosis [23–26]. Piezo1 is widely distributed throughout the body, especially in load-bearing tissues in the musculoskeletal system [27–29], while Piezo2 was mainly expressed in sensory neurons and tactile-sensory cutaneous Merkel cells [30, 31]. In IVD, Piezo1 has been verified to be expressed in NP and CEP [32–34]; Piezo1 activation is closely associated with mechanical loading-elicited apoptosis in NP cells [35]. Consequently, there exists a certain possibility that excessive aberrant mechanical loading-induced AFCs apoptosis is mediated by Piezo1. However, no research has explored the expression of Piezo1 in AF and the role of Piezo1 in AFCs apoptosis so far.

Based on the evidence above, we hypothesized that aberrant mechanical loading may activate Piezo1 to promote AFCs apoptosis and aggravate IVDD. To test our hypothesis, the expression of Piezo1 and activation status was detected in human and rat AF tissues and cultured AFCs. Then the CMS-stimulated rat AFCs apoptosis model and a lumbar instability model were employed to ascertain the effect of Piezo1 on AFCs. High-throughput sequencing and subsequent expression verification were utilized to elucidate the underlying mechanisms of CMS-induced AFCs apoptosis. This study is the first to verify the effects of Piezo1 on CMS-induced AFC apoptosis. It may help to better understand the relationship between Piezo1, aberrant mechanical loading, and AFC apoptosis during IVDD, which may provide a prospective strategy for IVDD therapy.

## Methods and materials

### In vivo experiments

#### Animal model

Three-month-old male Sprague Dawley rats were provided by the animal center of Xinqiao Hospital, Army Medical University. The lumbar instability model was based on the previously described methods [17]. Briefly, rats were anesthetized with 2% pentobarbital (50 mg/kg) and carprofen (5 mg/kg) for analgesia. The location of the L4/L5 disc was performed according to a previous study [36]. A line was drawn connecting the iliac crests, indicating the approximate level of the L5/L6 disc space, then L4 and L5 were located by palpation and labeled. A median incision was made in the posterior lumbar spine to expose the L4 and L5. The paraspinal muscle tissue was removed from L4–L5 to remove the dynamic forces created by the muscle forces transmitted across the spine, all lumbar spinous processes and posterior medial 1/2 of bilateral lumbar facet joints were removed, and the supraspinal and interspinous ligaments were excised with the scalpel to remove the static forces created by the bony and ligamentous architecture of the spine. Unbalanced dynamic and static forces were induced to reduce the posterior column stability to promote IVDD. After washing with normal saline containing 100 U/mL of penicillin, the wound was closed and the surface was disinfection again. In the control group, only a median incision was made and then sutured. Cefuroxime (30 mg/kg) was given for three days after the operation.

#### Magnetic resonance imaging

Four weeks after surgery, the rats were euthanized and the lumbar tissues were obtained. A magnetic resonance imager (PHILIPS Ingenia 3.0 T) was used to scan the lumbar tissue of the rats. The parameters are set as follows: TR time (2000 ms), TE time (80 ms), incentive times (2), scan time (3 min and 20 s), fat reduction technology (SPAIR), scan matrix (frequency encoding 368, phase encoding 288), layer thickness (2.5 mm), echo chains (12), and spin echo sequence (TSE sequence). Pfirrmann grading of rat intervertebral discs were calculated according to the Pfirrmann grading criteria.

#### Tissue specimen section

The vertebral body of L4 and L5 and the whole IVDD tissue were collected and fixed with 4% paraformaldehyde for 24 h. Tissue samples were decalcified through 10% EDTA decalcification solution (E1171, Sorlabio, Bejing, China) for 3 weeks. Then, the samples were dehydrated with ethanol solutions and embedded in liquid paraffin to make the paraffin sections. The specimens were then cut into 6-μm sections and placed on slides for later use.

#### Hematoxylin–eosin (H&E) staining and Sirius red staining

H&E staining was performed according to the standardized protocol. The histological grading scale system (Supplementary Table 1) included five categories with scores ranging from 0 points (normal) to 15 points (serious degeneration disc) using the method according to previous studies [37, 38]. The picrosirius red staining method of Novais et al. [39] and Melrose et al. [40] was used after the initial removal of tissue proteoglycans by pre-digestion with bovine testicular hyaluronidase (1000 U/ml) for 2 h at 37℃. The slides were initially stained in Wiegert’s iron hematoxylin for 30 min and stained in 0.1% Sirius red F3B (26–10-8, Sigma-Aldrich) in saturated aqueous picric acid for 2 h. The slides were then dehydrated in three changes of 100% ethanol, then cleared in xylene, and mounted in an Eukitt mounting medium. The sides were examined under polarized light through the microscope (BX53P, Olympus). The percentage of thin collagen fibers (green), intermediate collagen fibers (yellow), or thick collagen fibers (red) in AF was quantified by ImageJ software in the images (average pixels from three fields per section, three sections per rat, three rats per group). Color threshold levels were maintained constant between all analyzed images. Two observers evaluated the histological score of the intervertebral disc blindly.

#### Tunel staining

Sections were dewaxed and rehydrated. After incubation with 20 µg/mL proteinase K without DNase for 15 min, the sections were washed three times with PBS. Prepare Tunel staining solution (C1098, Beyotime, Shanghai, China) according to the reagent manufacturer’s instructions. The sections were incubated with working solution at 37 °C for 60 min in the dark. Sections were then incubated with 5 µg/mL Hoechst 33,258 solution (C1002, Beyotime) for 5 min and observed under the fluorescence microscope. Tunel-positive cells were counted in the images (average proportions from three fields per section, three sections per rat, three rats per group). The field size of regions of interest (ROI) was 400 μm × 700 μm.

### In vitro experiments

#### Isolation and culture of AFCs

AFCs preparation was based on the previously described methods [41]. A total of 32 rats was used to isolated AFCs in the in vitro cell experiments with a maximum of 4 independent repeat. Rats were euthanized by intraperitoneal injection of an overdose of sodium pentobarbital (150 mg/kg). AF tissue was isolated under sterile conditions and cut into approximately 1 mm^3^ fragments with ophthalmic scissors. For each isolation, the AF tissue of 8 rats was pooled and digested with 0.4% collagenase type II and 0.01% hyaluronidase type V for 90 min at 37 °C. Tissue debris was removed using a 70-μm cell filter, and the residues were then centrifuged at 400* g* for 5 min. The supernatant was discarded. Next, the sediment was resuspended by complete media containing Dulbecco’s modified Eagle medium /F-12 (C3130-0500, Viva Cell, Shanghai, China) and 10% fetal bovine serum (C2630-0500, Viva Cell), and 1% penicillin/streptomycin (C0222, Beyotime). Cells were cultured at 37 °C in a cell incubator containing 5% CO_2_. The medium was changed every 3 days, and the AFCs were passaged when the confluency reached 80–90%. The third generation of AFCs received different treatments including CMS, activator/inhibitor, and lentivirus, and the relevant experiments using these AFCs were regarded as one independent repeat.

#### Application of CMS

AFCs were seeded on Bioflex stretched 6-well plates (Flexcell International, Hillsborough, USA), and the cells were treated with CMS when the cell fusion rate reached 80–90%. The Flex-Cell 5000 tension system (Flexcell International) was used to simulate the CMS stimulation on AFCs. The parameter of CMS was selected as 0.5 Hz, 5–20% stretch deformation, 36 h duration, and the cycle mode was sinusoidal cycle, as reported in a previous study [42]; 20% is the maximum failure strain evaluated on annular collagen type I fibers, and 5% corresponds to a strain observed on the annular collagen type I fibers from intervertebral discs submitted to external stress. The loading frequency (0.5 Hz) and duration (36 h) can result in the apparent AFCs apoptosis without inducing cell detachment from the substrate or changing the cell phenotype [43]. After stretching for 36 h, cells were harvested for subsequent tests. Control groups were cultured similarly without CMS.

#### Chemical stimuli on Piezo1 channel

AFCs were treated with 1 μM Piezo1 special agonist Yoda1 (SML1558, Sigma-Aldrich, MO, USA) dissolved in DMSO to achieve chemical activation of Piezo1. AFCs were treated with 0.5 μM Piezo channel inhibitor GsMTx4 (HY-P1410, MCE, CA, USA) dissolved in DMSO to achieve chemical suppression of Piezo1. AFCs treated with DMSO were set as the control group.

#### Knockdown of Piezo1, Calpain1, and Calpain2

A Piezo1 shRNA lentiviral expression vector (pLVX-shRNA-Puro-Piezo1, Lv-Piezo1) and its control vector (pLVX-shRNA-Puro, Lv-ctrl) were constructed by XuanZun bioscience (Shanghai, China). The Piezo1 shRNA target sequence was 5’-GGAGTATGCCAACGAGAAGCA-3’. AFCs were inoculated with Lv-Piezo1 or Lv-ctrl for 24 h at a multiplicity of infection (MOI) of 20, respectively. Then, the AFCs were added with fresh medium. siRNA-Calpain1 and siRNA-Calpain2 were purchased from Tsingke Biotechnology (Beijing, China). To perform the knockdown experiments by siRNA, AFCs were transfected with siRNA Lipofectamine 2000 (11,668,030, Invitrogen, CA, USA) according to the manufacturer’s instructions.

#### Detection of Calpain activity

The detection of Calpain activity was performed using a Calpain activity assay (ab65308, Abcam, MA, USA) according to the manufacturer’s instructions. Briefly, 50 μg cleared supernatant was exposed to 5 μl calpain substrate for 1 h at 37 °C in the presence of a calpain reaction buffer. Fluorescence value was recorded at excitation of 400 nm and emission at 505 nm using a fluorescence plate reader. Relative fluorescence units were then calculated.

#### Apoptosis detection by flow cytometry

AFCs were digested with 0.05% trypsin EDTA (Hyclone, UT, USA) and harvested. Subsequently, Annexin V-PE/7-AAD Apoptosis Detection Kit (C1065L, Beyotime) was performed according to the manufacturer’s instructions. Cells were then evaluated with flow cytometry. Annexin V-PE + /7-AAD- cells (early apoptotic cells) and Annexin V-PE + /7-AAD + cells (late apoptotic cells) were considered as apoptotic cells. The apoptotic incidence was counted and expressed as a percentage of the total number of cells.

#### Apoptosis detection by Tunel staining assay

AFCs were washed three times with PBS and fixed with 4% paraformaldehyde for 30 min. Tunel assay solution (C1098, Beyotime) was prepared according to the reagent manufacturer’s instructions and incubated for 60 min in the dark. The nuclei were then stained with Hoechst 33,258. The samples were observed under the inverted fluorescence microscope (IX73, Olympus, Tokyo, Japan).

#### Apoptosis detection by MMP assay

MitoTracker Red CMXRos kit (C1049B, Beyotime) was used to detect the mitochondrial function of AFCs. Working solution was configured according to the reagent manufacturer’s instructions. AFCs were incubated with working solution at 37 °C for 30 min in the dark. Nuclei were stained with Hoechst 33,258. The samples were observed under the inverted fluorescence microscope.

#### Calcium ions influx detection

Intracellular calcium concentrations were measured by Fluo-4 AM (S1060, Beyotime). AFCs were seeded in small confocal laser dishes and were then loaded with 5 µM Fluo-4 AM for 1 h in Hank’s balanced salt solution containing 0.02% Pluronic F-127 (P6791, Solarbio, Beijing, China) and 1 mM Probenecid (SP9760, Solarbio, Beijing, China) at 37 °C for 1 h in darkness. Then, AFCs were washed 3 times with HBSS. The calcium influx was determined by laser confocal Ca^2+^ imaging technique to evaluate intracellular calcium transient in AFCs. Nuclei were counterstained with Hoechst 33,258.

#### Immunofluorescence staining assay

AFCs were fixed in 4% paraformaldehyde for 30 min. After incubation in immunostaining permeabilizer (P0096, Beyotime), AFCs were incubated with primary antibodies and overnight at 4 °C. Primary antibodies contain Piezo1 (1:100, 15,939–1-AP, Proteintech, Wuhan, China) and Piezo2 (1:500, PA5-72,976, Invitrogen, CA, USA). After washing three times with PBS, appropriate Goat Anti-Rabbit IgG(H + L) Dylight 488 or 648 fluorescent-labeled secondary antibody (1: 500, BS10034 and BS10017, Bioworld, Nanjing, China) was incubated for 1 h in darkness. Nuclei were counterstained with Hoechst 33,258 for 5 min. Images were captured by a laser confocal microscope.

#### Western blot test

AFCs were lysed with RIPA lysis buffer (P0013B, Beyotime), and the protein concentration was determined by BCA assay kit (PC0020, Solarbio); 1/5 volume of loading buffer was added to the lysate, and then the sample was placed in boiling water for 10 min. Proteins are separated by sulfate–polyacrylamide gel electrophoresis (SDS‒PAGE) techniques and transferred to the PVDF membrane. PVDF membrane was blocked by 5% skim milk for 1 h, and then the membrane was incubated with primary antibodies at 4° overnight. Primary antibodies contain calpain1 (10,538–1-AP, Proteintech), calpain2 (11,472–1-AP, Proteintech), Bax (50,599–2-Ig, Proteintech), cleaved-Caspase 3 (9664, Cell Signaling Technology, MA, USA), Piezo1 (5939–1-AP, Proteintech), and Gapdh (60,004–1-Ig, Proteintech). PVDF membrane was washed by TBST and then was incubated with the secondary antibody for 1 h. The secondary antibodies contain Goat Anti-Mouse IgG (H + L) HRP (abs20001, Absin, Shanghai, China) or Goat Anti-Rabbit IgG (H + L) HRP (abs20002, Absin). The expression of the target protein was detected by Clarity Western ECL Subs (1705060SP, Bio-Rad, Universal Hood III, CA, USA) and ChemiDoc imaging system (Bio-Rad) and analyzed by ImageJ software. All results were quantified and normalized to GAPDH.

#### High-throughput RNA-seq and data analysis

High-throughput RNA-seq was performed by Bioguoke Biotechnology Co., Ltd. (Beijing, China). Total RNA was extracted on ice using Trizol (15,596,018, ThermoFisher, DE, USA), RNA concentration and purity were measured using NanoDrop 2000 (ThermoFisher), and RNA integrity was assessed using the RNA Nano 6000 Assay Kit of the Agilent Bioanalyzer 2100 system (Agilent Technologies, CA, USA). Then, a cDNA library was assessed on the Agilent Bioanalyzer 2100 system. The Illumina Novaseq platform was utilized for high-throughput RNA-seq. After that, clean data were acquired by removing reads containing adapter, ploy-N, and low-quality reads from raw data. Transcripts were reconstructed using StringTie and HISAT2 tools software were utilized to map the clean data to reference rattus norvegicus genome. The expression of gene was analyzed by fragments per kilobase of transcript per million fragments mapped (FPKM). Differential expression genes were evaluated by the DESeq R package (1.10.1). The corrected *p*-value (FDR < 0.05) and |log2foldchange| (|FPKM|≥ 1) were regarded as the threshold for significant difference of gene expression. Finally, KOBAS software was used to perform the statistical enrichment of differential expression genes in Gene Ontology (GO), Kyoto Encyclopedia of Genes and Genomes (KEGG) pathways, and Gene Set Enrichment Analysis (GSEA).

#### Statistical analysis

Each experiment results were calculated from three biological repeats in technical triplicates. Statistical analysis was performed using GraphPad Prism 9 software (San Diego, CA, USA). All data were expressed as the mean ± standard deviation (mean ± SD). Two-group comparisons was evaluated using *t*-test. Multiple comparisons were evaluated using one-way ANOVA followed by Bonferroni’s post hoc test. *p* < 0.05 was considered as statistically significant.

## Results

### Lumbar instability promoted AFCs apoptosis and Piezo1 expression

**Fig. 1:**
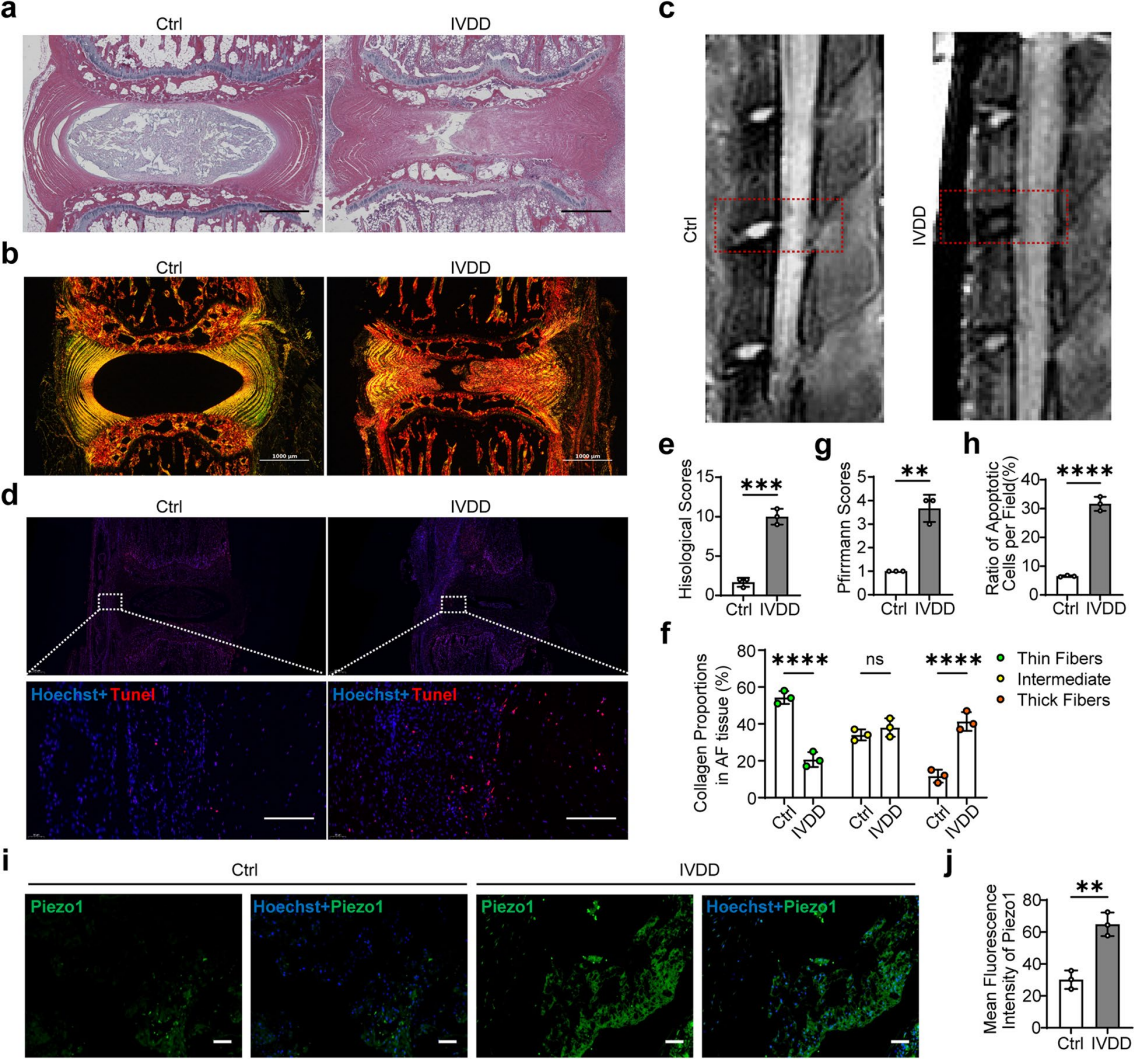
Lumbar instability promoted AFCs apoptosis and Piezo1 expression. **a** Representative H&E staining and histological scoring of rats in the Ctrl group and IVDD group (scale bar = 600 μm). **b** Representative Sirius red staining observed by polarized light microscopy of rats in the Ctrl group and IVDD group (scale bar = 1000 μm). **c** Representative MRI detection of the L4/L5 disc (red dotted box) of rats in the Ctrl group and IVDD group. **d** Representative Tunel staining (red) of AF tissue of rats in the Ctrl group and IVDD group (scale bar = 100 μm). Nuclei were counterstained with Hoechst 33,358 (blue). **e** Histological scoring of H&E staining results of rats in the Ctrl group and IVDD group (*n* = 3). **f** The percentage of thin collagen fibers (green), intermediate collagen fibers (yellow), or thick collagen fibers (red) in AF of rats in the Ctrl group and IVDD group (*n* = 3). **g** Pfirrmann grades of rats shown by MRI in the Ctrl group and IVDD group (*n* = 3). **h** Ratio of apoptotic cells of AF tissue of rats shown by Tunel staining in the Ctrl group and IVDD group (*n* = 3). **i** Representative immunofluorescent staining pictures detecting the expression of Piezo1 channel in AF tissue of the Ctrl group and IVDD group (scale bar = 150 μm). Piezo1 appeared green and nuclei were counterstained with Hoechst 33,358 (blue). **j** Statistic data of mean Piezo1 fluorescent intensity in AF tissue of the Ctrl group and IVDD group (*n* = 3). ^**^P < 0. 01, ^***^P < 0.001, ^****^P < 0.0001

Four weeks after the lumbar instability surgery, H&E staining of the L4/L5 disc and adjacent endplates revealed a normal, well-organized AF tissue and NP tissue in the control group with a lower histological score, whereas the IVDD group exhibited disorganized laminar structure, fragmented fibers and local fissures in AF tissue, and sharply decreased size in NP tissue with a higher histological score (Fig. 1a, e). The Sirius red staining results showed that the percentage of thick fibers was distinctly elevated in the IVDD group compared with control group, and the percentage of thin fibers was reduced in the IVDD group compared with control group (Fig. 1b, f). MRI results showed a lower signal and a higher Pfirrmann grading score in the L4/L5 disc in the IVDD group (Fig. 1c, g). These results indicated the successful establishment of IVDD model. TUNEL staining showed that the number of apoptotic cells increased significantly in AF tissue of IVDD rats (Fig. 1d, h). We found the expression of Piezo1 elevated in the AF tissue of IVDD rats (Fig. 1i, j), and the expression of Piezo2 is not distinct in AF tissue (Fig S1a). These results above indicated aberrant mechanical loading induced IVDD in rats, with elevated AFCs apoptosis and Piezo1 expression.

### CMS induced AFCs apoptosis and Piezo1 activation in vitro

**Fig. 2:**
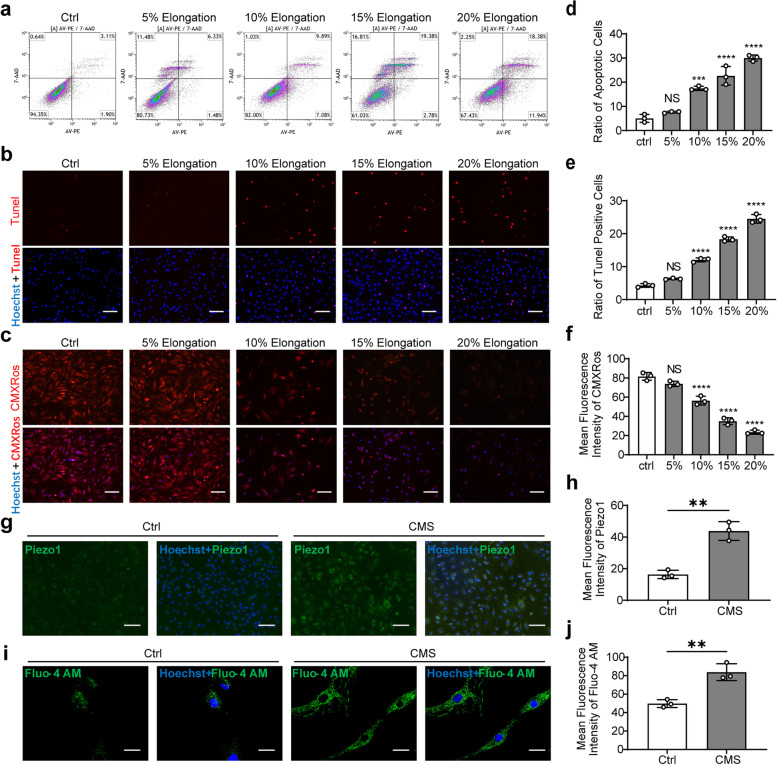
CMS-induced apoptosis of AFCs in vitro. **a** Representative flow cytometry scatter plots detecting AFCs apoptosis under different degrees of CMS. **b** Representative Tunel staining fluorescence pictures detecting AFCs apoptosis under different degrees of CMS (scale bar = 50 μm). Apoptotic AFCs appeared red and nuclei were counterstained with Hoechst 33,358 (blue). **c** Representative MMP staining fluorescence pictures detecting AFCs apoptosis under different degrees of CMS (scale bar = 50 μm). MMP appeared red and nuclei were counterstained with Hoechst 33,358 (blue). **d** Statistic data of apoptotic AFCs under different degrees of CMS determined by flow cytometry (*n* = 3). **e** Statistic data of Tunel-positive apoptotic AFCs under different degrees of CMS determined by Tunel staining (*n* = 3). **f** Statistic data of mean MMP fluorescent intensity in AFCs under different degrees of CMS determined by MMP staining (*n* = 3). **g** Representative immunofluorescent staining pictures detecting the expression of Piezo1 channel in AFCs of the Ctrl group and CMS group (scale bar = 50 μm). Piezo1 appeared green and nuclei were counterstained with Hoechst 33,358 (blue). **h** Statistic data of mean Piezo1 fluorescent intensity in AFCs of the Ctrl group and CMS group (*n* = 3). **i** Representative Fluo-4 AM staining pictures detecting the Ca^2+^ influx in AFCs of the Ctrl group and CMS group (scale bar = 10 μm). Fluo-4 AM appeared green and nuclei were counterstained with Hoechst 33,358 (blue). **j** Statistic data of mean Fluo-4 AM fluorescent intensity in AFCs of the Ctrl group and CMS group (*n* = 3). ^**^P < 0. 01, ^***^P < 0.001, ^****^P < 0.0001

Rats AFCs were imposed with CMS induced by Flexcell-5000 tension system. The total percentage of apoptotic AFCs including early apoptosis and late apoptosis significantly increased with the elevation of the extent of elongation, which peaked at 20% elongation (Fig. 2a, d). Moreover, similar results were also shown in the TUNEL staining (Fig. 2b, e). MMP assay results showed that with the increase of elongation, the MMP intensity gradually declined (Fig. 2c, f). These results showed that CMS could induce apoptosis of AFCs; 20% elongation induced the most distinct cellular apoptosis and was used in subsequent experiments. The expression of Piezo1 was upregulated in AFCs under CMS (Fig. 2g, h), and the expression of Piezo2 is not distinct in AFCs (Fig S1b). Calcium ions influx detection results showed that CMS promoted the Ca2 + influx in AFCs (Fig. 2i, j). These results suggested that Piezo1 was expressed in AFCs, and aberrant mechanical loading activated Piezo1 in AFCs in vivo and in vitro.

### Regulating the activation of Piezo1 affected AFCs apoptosis under CMS

**Fig. 3:**
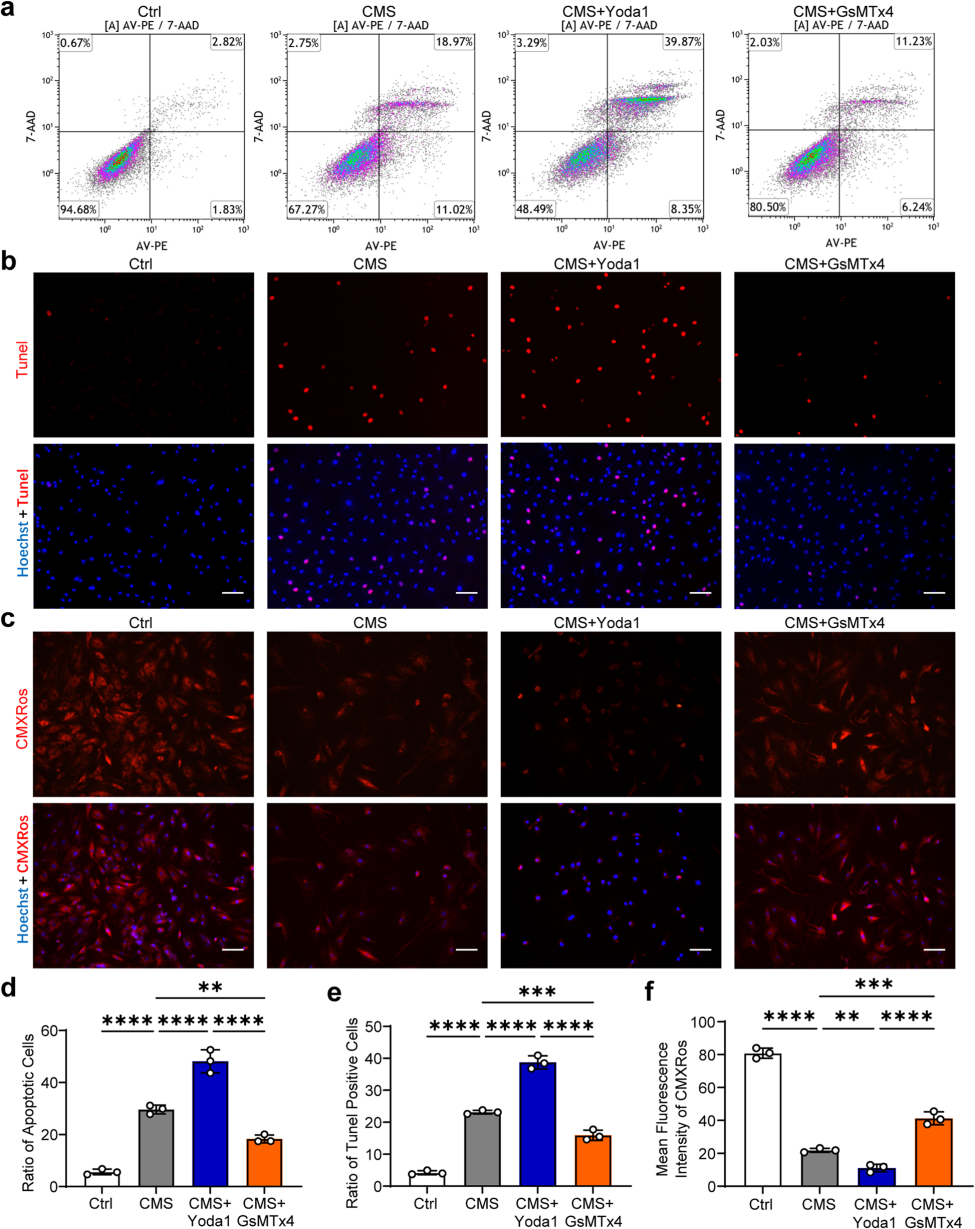
Yoda1 enhanced the apoptosis of AFCs, while GsMtx4 inhibited the apoptosis induced by CMS. **a** Representative flow cytometry scatter plots detecting AFCs apoptosis in the Ctrl group, CMS group, CMS + Yoda1 group, and CMS + GsMtx4 group. **b** Representative Tunel staining fluorescence pictures detecting AFCs apoptosis in the Ctrl group, CMS group, CMS + Yoda1 group, and CMS + GsMtx4 group (scale bar = 50 μm). Apoptotic AFCs appeared red and nuclei were counterstained with Hoechst 33,358 (blue). **c** Representative MMP staining fluorescence pictures detecting AFCs apoptosis in the Ctrl group, CMS group, CMS + Yoda1 group, and CMS + GsMtx4 group (scale bar = 50 μm). MMP appeared red and nuclei were counterstained with Hoechst 33,358 (blue). **d** Statistic data of apoptotic AFCs in the Ctrl group, CMS group, CMS + Yoda1 group, and CMS + GsMtx4 group determined by flow cytometry (*n* = 3). **e** Statistic data of Tunel-positive apoptotic AFCs in the Ctrl group, CMS group, CMS + Yoda1 group, and CMS + GsMtx4 group determined by Tunel staining (*n* = 3). **f** Statistic data of mean MMP fluorescent intensity in AFCs in the Ctrl group, CMS group, CMS + Yoda1 group, and CMS + GsMtx4 group determined by MMP staining (*n* = 3). ^**^P < 0. 01, ^***^P < 0.001, ^****^P < 0.0001

To further explore the mechanism of Piezo1 in AFCs apoptosis, Yoda1 and GsMtx4 were applied in AFCs under CMS, respectively. Flow cytometry analysis showed that Yoda1 enhanced AFCs apoptosis, while GsMtx4 alleviated AFCs apoptosis (Fig. 3a, d). Similar results were also shown in the Tunel staining (Fig. 3b, e). MMP assay results showed that Yoda1 aggravated the MMP intensity reduction while GsMtx4 ameliorated the MMP intensity reduction stimulated by CMS (Fig. 3c, f). These results indicated that the activation status of Piezo1 is associated with AFCs apoptosis under CMS.

### Knocking down Piezo1 reduced AFCs apoptosis under CMS

**Fig. 4:**
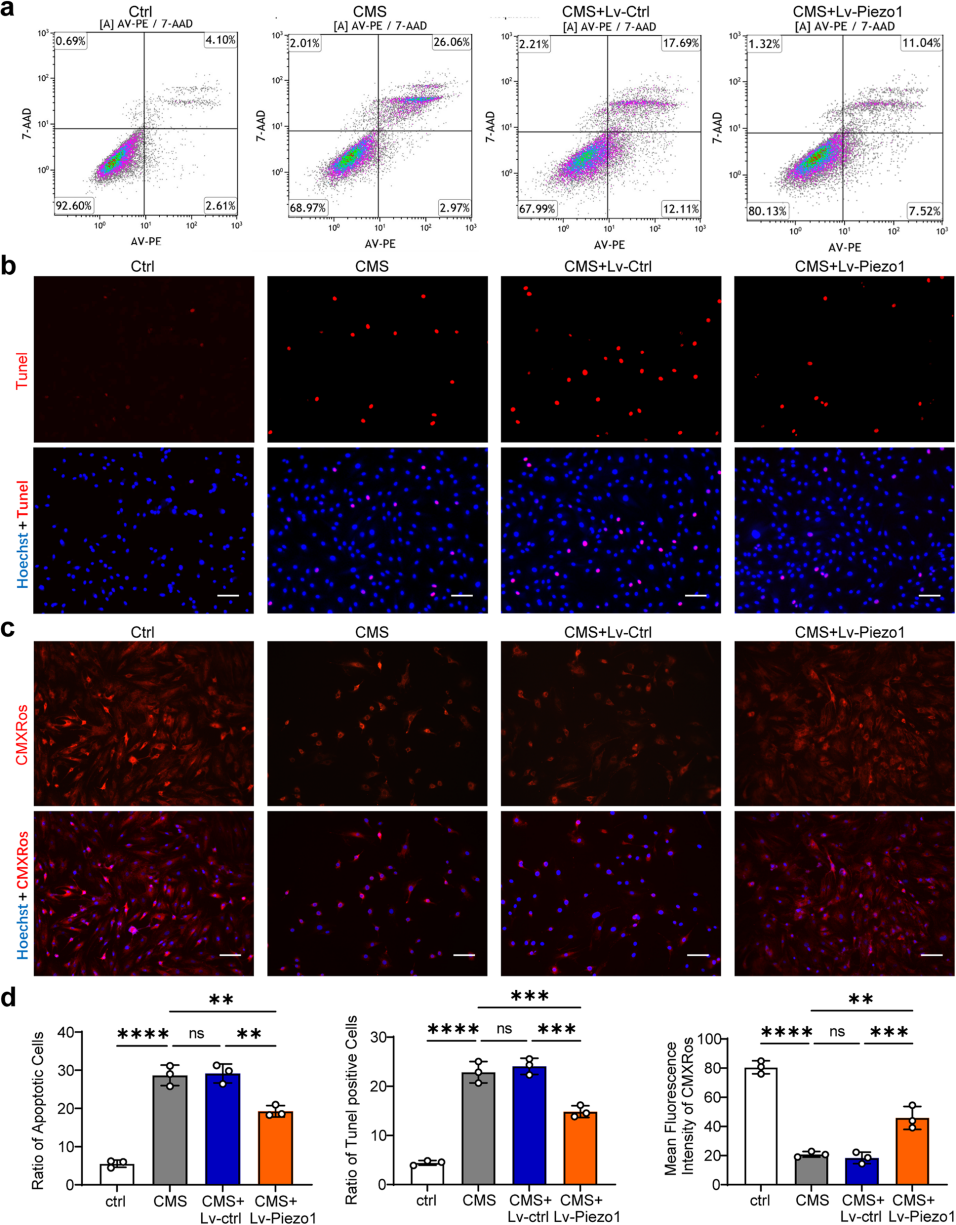
Knockdown of Piezo1 reduced the apoptosis induced by CMS. **a** Representative flow cytometry scatter plots detecting AFCs apoptosis in the Ctrl group, CMS group, CMS + Lv-Ctrl group, and CMS + Lv-Piezo1 group. **b** Representative Tunel staining fluorescence pictures detecting AFCs apoptosis in the Ctrl group, CMS group, CMS + Lv-Ctrl group, and CMS + Lv-Piezo1 group (scale bar = 50 μm). Apoptotic AFCs appeared red and nuclei were counterstained with Hoechst 33,358 (blue). **c** Representative MMP staining fluorescence pictures detecting AFCs apoptosis in the Ctrl group, CMS group, CMS + Lv-Ctrl group, and CMS + Lv-Piezo1 group (scale bar = 50 μm). MMP appeared red and nuclei were counterstained with Hoechst 33,358 (blue). **d** Statistic data of apoptotic AFCs in the Ctrl group, CMS group, CMS + Lv-Ctrl group, and CMS + Lv-Piezo1 group determined by flow cytometry (*n* = 3). **e** Statistic data of Tunel-positive apoptotic AFCs in the Ctrl group, CMS group, CMS + Lv-Ctrl group, and CMS + Lv-Piezo1 group determined by Tunel staining (*n* = 3). **f** Statistic data of mean MMP fluorescent intensity in AFCs in the Ctrl group, CMS group, CMS + Lv-Ctrl group, and CMS + Lv-Piezo1 group determined by MMP staining (*n* = 3). ^**^P < 0.01, ^***^P < 0.001, ^****^P < 0.0001

To further confirm the role of Piezo1 in CMS-induced AFCs apoptosis, LV-Piezo1 was used to knock down the expression of Piezo1 in AFCs, and the effectiveness of shRNA was verified (Fig. S2a, b). Flow cytometry showed that the apoptosis rate of the Lv-Piezo1 group was reduced compared with the Lv-Ctrl group and CMS group (Fig. 4a, d). Tunel staining showed that the Tunel-positive cells in the Lv-Piezo1 group was lesser than that in the control group and Lv-Ctrl group (Fig. 4b, e). MMP assay results showed that knockdown Piezo1 alleviated MMP intensity reduction under CMS (Fig. 4c, f). These results indicated that Piezo1 mediated the apoptosis of AFCs stimulated by CMS.

### RNA-seq showed Piezo1-mediated AFCs apoptosis via calcium signaling pathway

**Fig. 5:**
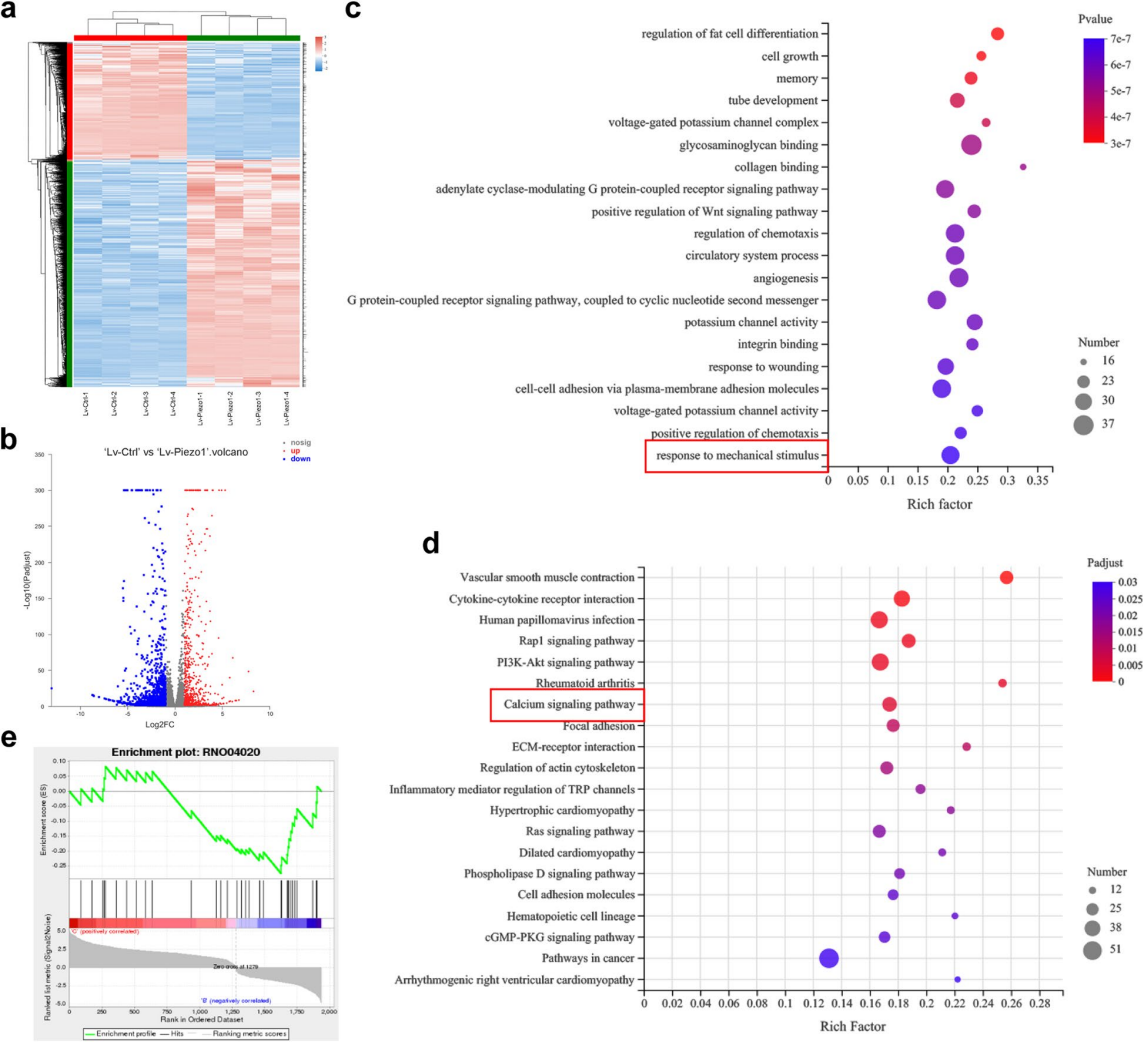
CMS affected the Calcium signaling pathway in AFCs. **a** Heatmap of differentially expressed genes in AFCs treatment with Lv-Ctrl and Lv-Piezo1 under CMS (*n* = 4). **b** Volcano map of differentially expressed genes in AFCs treatment with Lv-Ctrl and Lv-Piezo1 under CMS. **c** GO pathway analysis of differentially expressed genes in AFCs treatment with Lv-Ctrl and Lv-Piezo1 under CMS. **d** KEGG pathway analysis of differentially expressed genes in AFCs treatment with Lv-Ctrl and Lv-Piezo1 under CMS. **e** GSEA analysis of Calcium signaling pathway (KEGG pathway number: RNO04020) in AFCs treatment with Lv-Ctrl and Lv-Piezo1 under CMS

RNA-seq was performed in 4 in vitro experimental biological repeats received Lv-Ctrl + CMS treatment or Lv-Piezo1 + CMS treatment. Then 4 samples of Lv-Ctrl treated AFCs under CMS and 4 samples of Lv-Piezo1 treated AFCs under CMS were acquired, respectively. Then RNA-seq was performed to further explore the underlying mechanisms by which Piezo1 affects AFCs apoptosis. Heatmap and volcano plot exhibited the differentially expressed genes (Fig. 5a, b). GO analysis showed that the differentially expressed genes were significantly enriched in “response to mechanical stimulus” (Fig. 5c). KEGG pathway analysis revealed that the differentially expressed genes were significantly enriched in “calcium signaling pathway” (Fig. 5d). GSEA analysis showed that Lv-Piezo1-mediated Piezo1 suppression was closely associated with the calcium signaling pathway downregulation in AFCs (Fig. 5e). These results revealed that Piezo1 may affect the function of AFCs by calcium signaling pathway.

### Piezo1 promotes AFCs apoptosis via Ca2 + /Calpain2/Caspase3 pathway

**Fig. 6:**
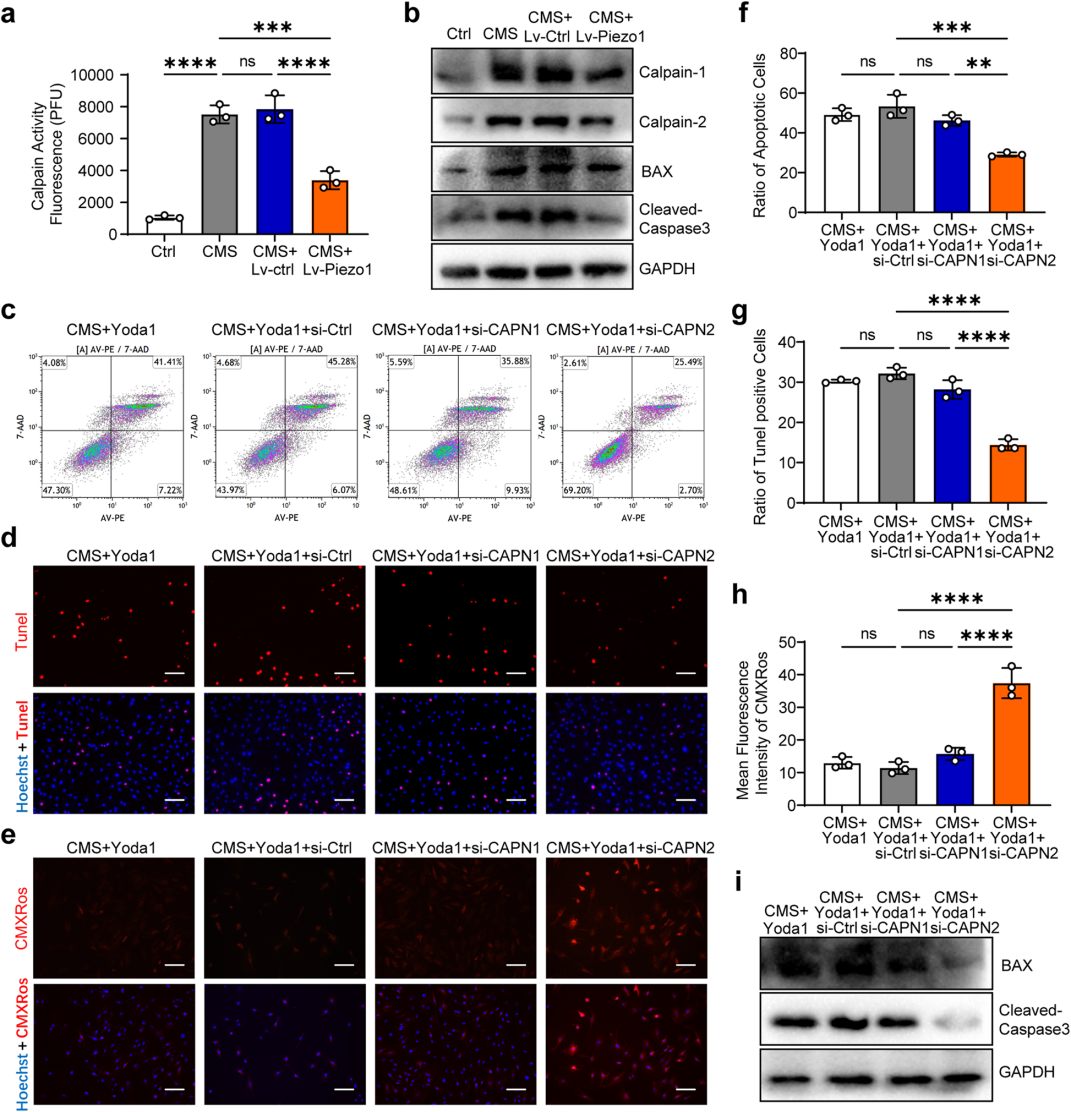
Piezo1 promotes AFCs apoptosis via Ca2 + /Calpain2/Caspase3 pathway. **a** The activity of Calpains in the Ctrl group, CMS group, CMS + Lv-Ctrl group, and CMS + Lv-Piezo1 group as determined by Calpain activity detection kit (*n* = 3). **b** Western blotting analysis showing the Calpain1, Calpain2, Bax, Cleaved-Caspase3 expression in the Ctrl group, CMS group, CMS + Lv-Ctrl group, and CMS + Lv-Piezo1 group (*n* = 3). **c** Representative flow cytometry scatter plots detecting AFCs apoptosis in the CMS + Yoda1 group, CMS + Yoda1 + si-Ctrl group, CMS + Yoda1 + si-CAPN1 group, and CMS + Yoda1 + si-CAPN2 group. **d** Representative Tunel staining fluorescence pictures detecting AFCs apoptosis in the CMS + Yoda1 group, CMS + Yoda1 + si-Ctrl group, CMS + Yoda1 + si-CAPN1 group, and CMS + Yoda1 + si-CAPN2 group (scale bar = 50 μm). Apoptotic AFCs appeared red and nuclei were counterstained with Hoechst 33,358 (blue). **e** Representative MMP staining fluorescence pictures detecting AFCs apoptosis in the CMS + Yoda1 group, CMS + Yoda1 + si-Ctrl group, CMS + Yoda1 + si-CAPN1 group, and CMS + Yoda1 + si-CAPN2 group (scale bar = 50 μm). MMP appeared red and nuclei were counterstained with Hoechst 33,358 (blue). **f** Statistic data of apoptotic AFCs in the CMS + Yoda1 group, CMS + Yoda1 + si-Ctrl group, CMS + Yoda1 + si-CAPN1 group, and CMS + Yoda1 + si-CAPN2 group determined by flow cytometry (*n* = 3). **g** Statistic data of Tunel-positive apoptotic AFCs in the CMS + Yoda1 group, CMS + Yoda1 + si-Ctrl group, CMS + Yoda1 + si-CAPN1 group, and CMS + Yoda1 + si-CAPN2 group determined by Tunel staining (*n* = 3). **h** Statistic data of mean MMP fluorescent intensity in AFC in the CMS + Yoda1 group, CMS + Yoda1 + si-Ctrl group, CMS + Yoda1 + si-CAPN1 group, and CMS + Yoda1 + si-CAPN2 group determined by MMP staining (*n* = 3). **i** Western blotting analysis showing the Bax, Cleaved-Caspase3 expression in the CMS + Yoda1 group, CMS + Yoda1 + si-Ctrl group, CMS + Yoda1 + si-CAPN1 group, and CMS + Yoda1 + si-CAPN2 group (*n* = 3). ^**^P < 0. 01, ^***^P < 0.001, ^****^P < 0.0001

Next, we explored the downstream mechanism in Piezo1-mediated apoptosis of AFCs. Piezo1-activated calcium signaling pathway can induce downstream calpains activation, which is closely related to apoptosis. We speculated that Piezo1-activated calcium signaling pathway may regulate AFCs apoptosis through calpains. Calpain activity detection kit results showed that CMS elevated the activity of Calpains, and Lv-Piezo1 treatment reduced the activity of Calpains (Fig. 6a). Western blot results showed that the expression of Calpain1, Calpain2, and downstream Bax, Cleaved-Caspase3 were all elevated after CMS and declined after Lv-Piezo1 treatment (Fig. 6b and Fig. S3a-d). To specify the certain Calpain that underlies Piezo1-mediated AFCs apoptosis, siRNA-Calpain1 and siRNA-Calpain2 were used to inhibit the activation of Calpain1 and Calpain2 in Yoda1-treated AFCs under CMS, respectively (Fig. S2b-e). Flow cytometry, Tunel staining, and MMP staining results showed that apoptosis of AFCs was inhibited after siRNA-Calpain2 treatment but not after siRNA-Calpain1 treatment (Fig. 6c-h). Western blot results showed that the expression of Bax and Cleaved-Caspase3 were suppressed after siRNA-Calpain2 treatment (Fig. 6i and Fig. S3e-f). These results indicated that CMS increases AFCs apoptosis by activating Piezo1 and Ca2 + /Calpain2/Caspase3 pathway.

### Knocking down Piezo1-alleviated AFCs apoptosis and IVDD in rats

**Fig. 7:**
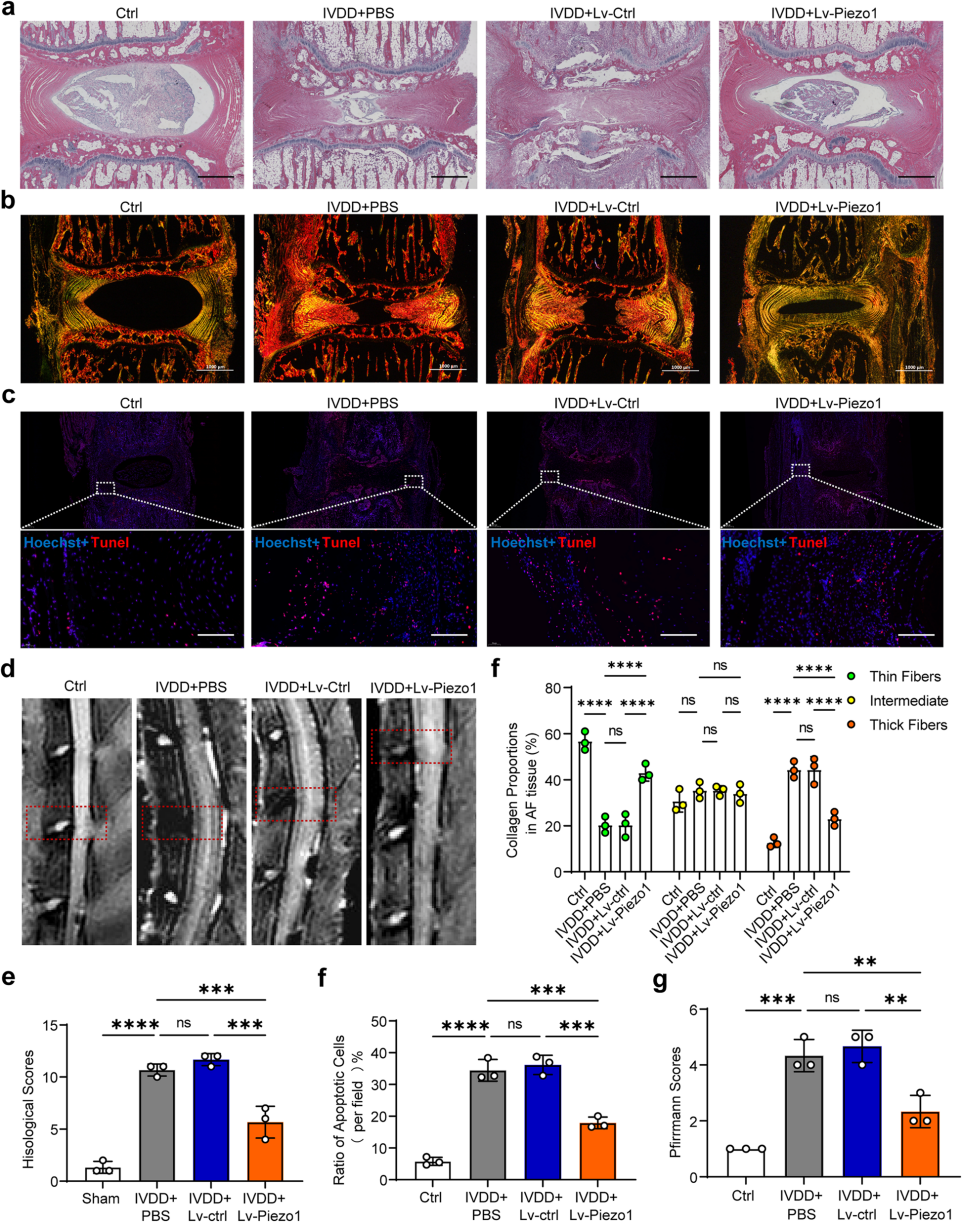
Knockdown of Piezo1 alleviated the lumbar instability induced IVDD in rats. **a** Representative H&E staining of rats in the Ctrl group, IVDD + BPS group, IVDD + Lv-Ctrl group, and IVDD + Lv-Piezo1 group. (scale bar = 600 μm). **b** Representative Sirius red staining observed by polarized light microscopy of rats in the Ctrl group, IVDD + BPS group, IVDD + Lv-Ctrl group, and IVDD + Lv-Piezo1 group (scale bar = 1000 μm). **c** Representative MRI detection of the L4/L5 disc (red dotted box) of rats in the Ctrl group, IVDD + BPS group, IVDD + Lv-Ctrl group, and IVDD + Lv-Piezo1 group. **d** Representative Tunel staining (red) of AF tissue of rats in the Ctrl group, IVDD + BPS group, IVDD + Lv-Ctrl group, and IVDD + Lv-Piezo1 group (scale bar = 100 μm). **e** Histological scoring of H&E staining results of rats in the Ctrl group, IVDD + BPS group, IVDD + Lv-Ctrl group, and IVDD + Lv-Piezo1 group (*n* = 3). **f** The percentage of thin collagen fibers (green), intermediate collagen fibers (yellow), or thick collagen fibers (red) in AF of rats in the Ctrl group, IVDD + BPS group, IVDD + Lv-Ctrl group, and IVDD + Lv-Piezo1 group (*n* = 3). **g** Pfirrmann grades of rats shown by MRI in the Ctrl group, IVDD + BPS group, IVDD + Lv-Ctrl group, and IVDD + Lv-Piezo1 group (*n* = 3). **h** Ratio of apoptotic cells of AF tissue of rats shown by Tunel staining in the Ctrl group, IVDD + BPS group, IVDD + Lv-Ctrl group, and IVDD + Lv-Piezo1 group (*n* = 3). ^**^P < 0. 01, ^***^P < 0.001, ^****^P < 0.0001

**Fig. 8:**
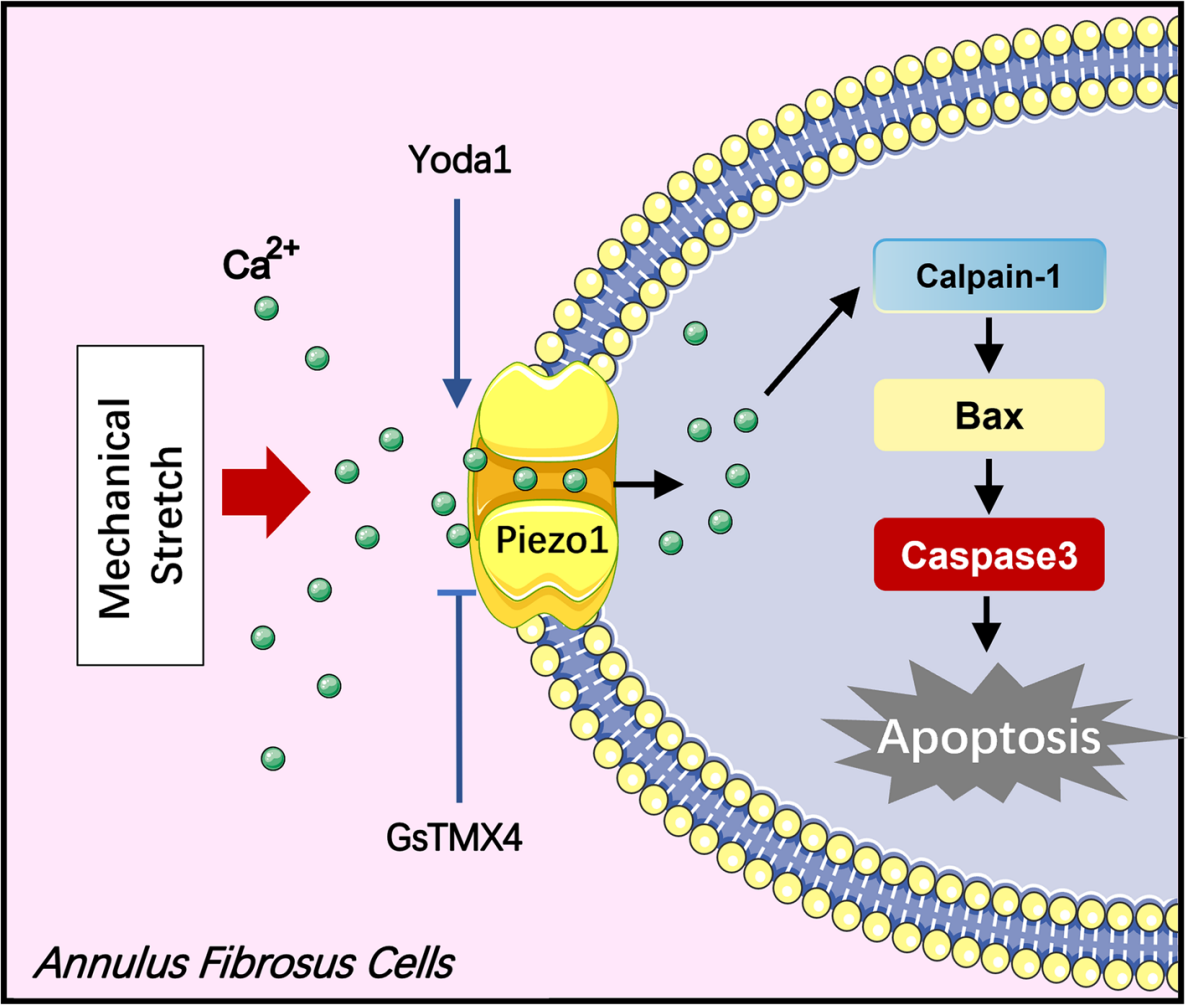
Schematic diagram of aberrant mechanical loading-induced AFCs apoptosis via activating Piezo1 channel

H&E staining of the L4/L5 disc in Lv-Piezo1 treated IVDD rats revealed that Lv-Piezo1 treatment alleviated the disorganized laminar structure AF tissue and decreased size in NP tissue, which resulted in elevated histological score compared with Lv-Ctrl or PBS treated IVDD rats (Fig. 7a,e). Sirius red staining results showed that Lv-Piezo1 treatment reduced the percentage of thick fibers and elevated the percentage of thin fibers in IVDD rats (Fig. 7b, f). Tunel staining showed that the number of apoptotic cells declined in AF tissue of Lv-Piezo1 treated IVDD rats (Fig. 7c,g). MRI results showed Lv-Piezo1 treated IVDD rats obtained elevated Pfirrmann grading score in the L4/L5 disc compared with Lv-Ctrl- or PBS-treated IVDD rats (Fig. 7d, h). These results showed that Piezo1 knockdown inhibited AFCs apoptosis and IVDD in rats. Schematic diagram of aberrant mechanical loading-induced AFCs apoptosis via activating Piezo1 channel were shown in Fig. 8.

## Discussion

Excessive aberrant mechanical loading-induced AFCs apoptosis is an elicitation of IVDD. However, the underlying mechanisms by which aberrant mechanical loading promotes AFCs apoptosis are not clear. In this study, we explored the role of a mechanosensitive ion channel protein Piezo1 in aberrant mechanical loading-induced AFCs apoptosis and investigated the potential mechanisms. We found that the expression of piezo1 was elevated in the AF of rat experimental IVDD. CMS elicited distinct AFCs apoptosis, as well as up-regulated Piezo1 expression and Piezo1 channel activation. Yoda1 promoted AFCs apoptosis, while GsMtx4 and lentivirus-based Piezo1 knockdown suppressed AFCs apoptosis. RNA-seq showed that Piezo1 activated the calcium signaling pathway, which then activated downstream Calpain2/Bax/Caspase3 axis to stimulate AFCs apoptosis. Intradiscal administration of Lv-Piezo1 significantly alleviated the progress of IVDD in rat experimental IVDD. This study reveals the role of Piezo1 in aberrant mechanical loading stimulated AFCs apoptosis during IVDD and may facilitate the effects of current IVDD therapies.

As a crucial supporting component in the biomechanical properties of IVD, the structural and mechanical integrity of AF is crucial in confining NP. The AF is generally the first site to be injured in the onset of IVDD, after that scar tissue resulting from the healing of small tears or fissures can cause progressive weakening of IVD. Previous studies verified that AF injury is closely related to AFCs apoptosis. The reasons that induced AFCs apoptosis is complex; the excessive aberrant mechanical loading is a unique factor in promoting AFCs apoptosis, which is also proved to be closely related to the cellular apoptosis in other fibrous tissue like tendon, ligamentum flavum, and anterior cruciate ligament [44–46]. Surgery-induced unbalanced dynamic and static forces, which are elicited by lumbar instability surgery, are reported to result in excessive aberrant mechanical loading in AF and contributed to IVDD [47]. This lumbar instability model can cause distinct AFCs apoptosis, as shown in our Tunel staining results and previous studies [17, 18]. In vitro studies also proved that 20% elongation of CMS stimulated distinct apoptosis of AFCs, which is also similar to the cellular response of AFCs to CMS reported by Zhang et al. [42].

Piezo1 and Piezo2 are mechanically sensitive ion channel proteins that convert extracellular mechanical stimuli to intracellular signaling [48, 49]. Piezo1 and Piezo2 participate in aberrant mechanical loading-induced apoptosis in chondrocytes, type II pneumocytes, and tumor cells [50–52]. Piezo1 activation also stimulates the NPCs apoptosis during IVDD [35, 53, 54]. Therefore, we explored the expression of Piezo1 and Piezo2 in AF and investigated whether Piezo1 and Piezo2 mediated the aberrant mechanical loading-induced AFCs apoptosis. We found that Piezo1 is highly expressed in AF, whereas Piezo2 is barely expressed in AF. Using Piezo1-specific activator Yoda1, we found that Piezo1 activation enhanced the CMS-induced AFCs apoptosis. A mechanosensitive cation channels blocker GsMTx4 suppressed the CMS-induced AFCs apoptosis. However, GsMtx4 is not a specific Piezo1 inhibitor for it also hinders the activation of other mechanosensitive cation channels like TRPC1 and TRPC3 [55, 56]. Therefore, we accomplished Piezo1 knockdown via Lv-Piezo1 transfection and verified that Piezo1 activation enhanced the CMS-induced AFCs apoptosis. Moreover, imagological and histological results exhibited that intradiscal administration of Lv-Piezo1 alleviated lumbar instability induced IVDD. These results proved that Piezo1 is a modulator in CMS-induced AFCs apoptosis.

We then investigate the downstream intracellular signaling stimulated by the Piezo1 activation. RNA-seq and KEGG analysis exhibited that the calcium signaling pathway was enriched when the Piezo1 was knockdown in CMS-induced apoptotic AFCs. Piezo1 activation helps cells to convert mechanical signals into biological signals via promoting Ca2 + influx and altering subsequent intracellular calcium signaling to elicit a series of cellular biological processes [57, 58]. Ca2 + plays a central role as a second messenger in eukaryotic signal transduction [59]. The intracellular free Ca2 + concentration is maintained at lower levels than extracellular fluid [60]. Ca2 + overload is associated with all three apoptotic pathways, the intrinsic apoptotic pathway (the mitochondrial pathway), the extrinsic apoptotic pathway (the death receptor pathway), and the endoplasmic reticulum pathway [61, 62]. Our results found that Piezo1 activation promotes the expression of proapoptotic protein Bax and cysteine protease Caspase3. Ca2 + overload can trigger mitochondrial permeability transition via opening the permeability transition pore, then initiate the activation of consequent apoptogenic factors including Bax and initiate the caspase cascade reaction [63–65]. Previous studies have explored the potential mechanisms underlying piezo1 and Ca2 + -induced NPCs apoptosis. Mechanical compression activates Piezo1 in NPCs and induced apoptosis via Ca2 + -induced mitochondria dysfunction [35]. Stiff extracellular matrix activates Piezo1 channel and increased intracellular Ca2 + levels and promotes NPCs apoptosis via aggravating endoplasmic reticulum stress [53]. However, unlike NPCs, AFCs are under excessive aberrant mechanical loading elicited by direct radial tensile from the NP expansion and cranial–caudal stretch from the separation of the two endplates [9]. Therefore, the underlying mechanism that piezo1-induced AFCs apoptosis may be discrepant from piezo1-induced NPCs apoptosis.

Calpains are special Ca2 + -activated non-lysosomal cysteine proteases that are closely related to Ca2 + -mediated apoptosis via caspase activity [66, 67]. The activity of calpains is tightly regulated by intracellular Ca2 + concentration. Calpains consist of several isoforms. In these isoforms, conventional calpains are ubiquitously expressed across all tissues and organs including IVD [68, 69]. Conventional calpains have two isoforms: Calpain1 (µ-calpain) that requires micromolar Ca2 + for activation and Calpain2 (m-calpain) that requires millimolar Ca2 + for activation. Both calpain-1 and calpain2 are closely related to mechanical force-stimulated cell apoptosis [70–74]. Piezo1 has been reported to activate Calpain-2 through the elevated Ca2 + influx [50, 75], and Piezo1-specific activator Yoda1 can enhance Calpain-2 activation [76]. Our results found that CMS enhanced the expression of Calpain1 and Calpain2, and silencing Calpain2, not Calpain1 could reverse the Yoda1- and CMS-stimulated AFCs apoptosis, indicating that piezo1 promotes AFCs apoptosis via Ca2 + influx-mediated Calpain2 activation. To investigate the downstream effectors of Calpain-2 activation, we explored the expression of Bax that can be cleaved by Calpain-2 to induce the cellular apoptosis [77–80]. Our results found that CMS enhanced the expression of Bax and cleaved-Caspase3, whereas knocking down Piezo1 could reverse the CMS-induced AFCs apoptosis. Silencing Calpain2 could suppress the expression of Bax and cleaved-Caspase3, indicating that piezo1 promotes AFCs apoptosis via Calpain2/Bax/Caspase3 pathway.

Although the in vivo experiments using the lumbar instability model and in vitro experiments using the CMS-treated AFCs model proved that aberrant mechanical loading activates Piezo1 to induce AFCs apoptosis in IVDD, it should be noted that it cannot completely simulate the situation in IVDD given the complex biomechanics alteration during the progress of IVDD. For example, Bonnevie reported that the release of residual strain in the degenerative intervertebral disc is related to the AFCs apoptosis [7]. Besides, the aberrant compressive overload contributes to AFCs apoptosis and aggravates IVDD, as shown by the static or transient disc bending models [81, 82], and static or dynamic axis compressive models [83, 84]. In addition, asymmetric loading has been reported to elicit AFCs apoptosis and subsequent IVDD [8]. Moreover, aberrant shear stress is also a non-negligible biomechanical factor that can result in IVDD and AF tissue disruption, although no direct evidence demonstrates that aberrant shear stress can lead to AFCs apoptosis [85]. The unbalanced dynamic and static forces stimulated by the lumbar instability model can result in AFCs apoptosis and exacerbate IVDD, but we surmise it only accounts for a portion of aberrant mechanical loading-induced degenerative alteration and AFCs apoptosis. However, given that Piezo1 participates in the cellular response to a variety of mechanical loading including compression, stretch, and shear force, there exists a certain possibility that piezo1 responses to these aberrant mechanical loadings and participates in AFCs apoptosis and IVDD progression.

In summary, we explored the expression of Piezo1 in AF tissue and the role of Piezo1 in CMS-stimulated AFCs for the first time. We verified that aberrant mechanical loading could initiate AFCs apoptosis to promote IVDD, which is mediated by activating Piezo1 and subsequent calcium signaling pathway to active downstream Calpain2/Bax/Caspase3 axis. Knocking down Piezo1 could alleviate the progress of IVDD initiated by lumbar instability surgery. Therefore, Piezo1 is a crucial factor in inducing AFCs apoptosis and Piezo1 is expected to be a potential therapeutic target in treating IVDD.

## Conclusions

Piezo1 is highly expressed in AF tissue and AFCs. Aberrant mechanical loading could induce AFCs apoptosis by activating Piezo1 and downstream Calpain2/Bax/Caspase3 pathway to induce IVDD. Knocking down Piezo1 could effectively alleviate the progress of IVDD. Therefore, Piezo1 possesses the potential to become a therapeutic target in treating IVDD in the future.

## Supplementary Information

**Additional file 1: Figure S1.** The expression of Piezo2 in AF tissue and AFCs. (a) Representative immunofluorescent staining pictures detecting the expression of Piezo2 channel in AF tissue of the Ctrl group and IVDD group (scale bar = 150 μm). Piezo2 appeared green and nuclei were counterstained with Hoechst 33358 (blue). (b) Representative immunofluorescent staining pictures detecting the expression of Piezo2 channel in AFCs of the Ctrl group and CMS group (scale bar = 50 μm). Piezo2 appeared green and nuclei were counterstained with Hoechst 33358 (blue).** Figure S2.** Verification of Piezo1 knockdown by Lv-Piezo1 (*n* = 3). Verification of Calpain1 and Calpain2 knockdown by si-CAPN1 and siCAPN2 (*n* = 3). **Figure S3.** Statistic data of western blotting. (a-d) Western blotting analysis showing the Calpain1, Calpain2, Bax, Cleaved-Caspase3 expression in the Ctrl group, CMS group, CMS + Lv-Ctrl group, and CMS + Lv-Piezo1 group (*n* = 3). (e-f) Western blotting analysis showing the Bax, Cleaved-Caspase3 expression in the CMS + Yoda1 group, CMS + Yoda1 + si-Ctrl group, CMS + Yoda1 + si-CAPN1 group, and CMS + Yoda1 + si-CAPN2 group (*n* = 3). **P<0.01, ***P<0.001, ****P<0.0001.** Table S1. **Histological grading scale system.

## Data Availability

The datasets described and analyzed during the current study are available from the corresponding author on reasonable request. The RNA sequencing data used to support the findings of this study has been deposited in the Sequence Read Archive (https://www.ncbi.nlm.nih.gov/) repository (GSE216445).

## Abbreviations

AFCs: Annulus fibrosus cells
IVDD: Intervertebral disc degeneration
CMS: Cyclic mechanical stretch
MMP: Mitochondrial membrane potential
Lv-Piezo1: Lentiviral shRNA-Piezo1
RNA-seq: High-throughput RNA sequencing
LBP: Lower back pain
IVD: Intervertebral disc
NP: Nucleus pulposus
AF: Annulus fibrosus
CEP: Cartilaginous endplates
FPKM: Fragments mapped
GO: Gene Ontology
KEGG: Kyoto Encyclopedia of Genes and Genomes
GSEA: Gene Set Enrichment Analysis
