# Supplementary figures and images for: Aberrant mechanical loading induces annulus fibrosus cells apoptosis in intervertebral disc degeneration via mechanosensitive ion channel Piezo1
Source: Arthritis Res Ther. 2023 Jul 7;25:117. doi: 10.1186/s13075-023-03093-9 (PMC10327399; doi:10.1186/s13075-023-03093-9)

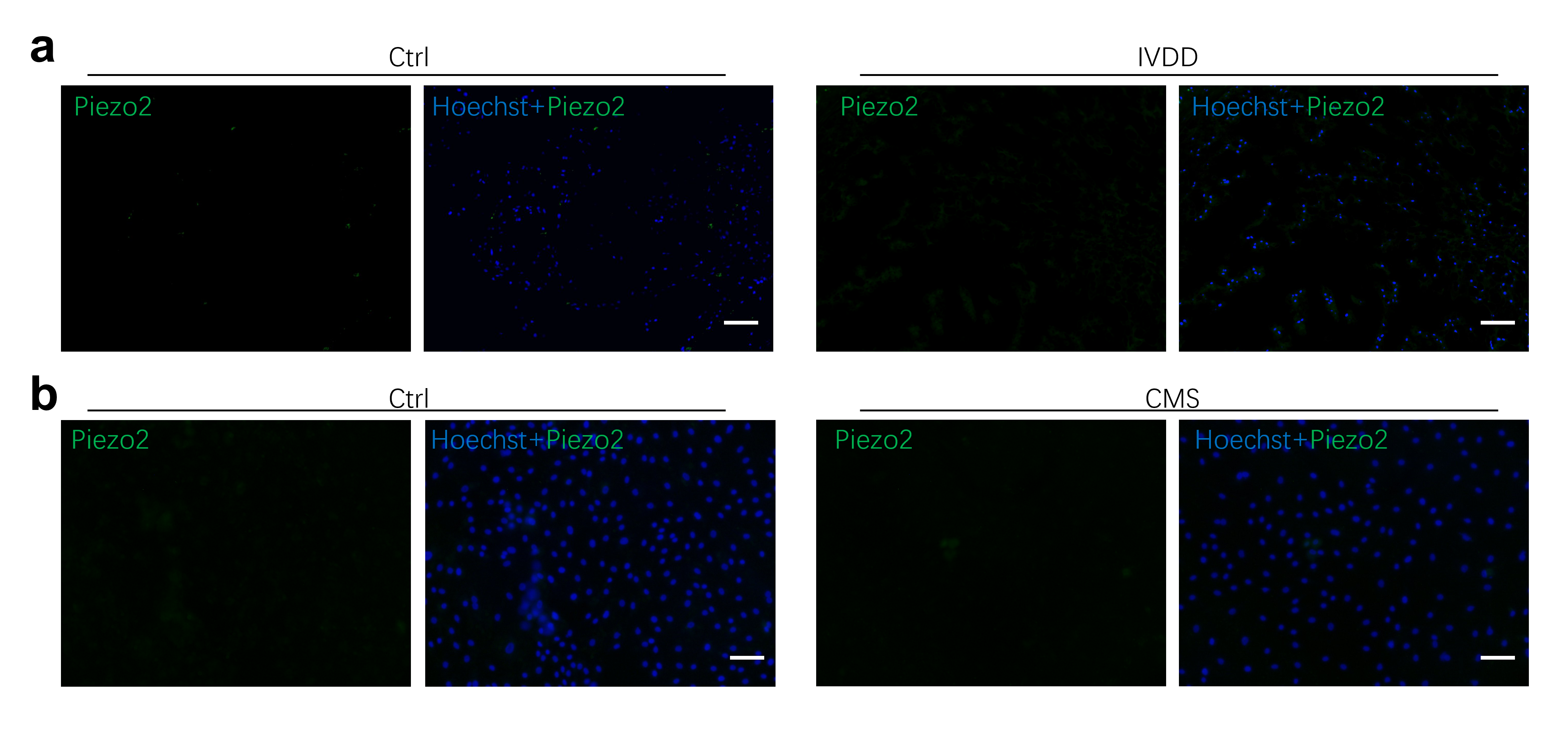

Supplement: Supplementary file 1 — Additional file 1: Figure S1. The expression of Piezo2 in AF tissue and AFCs. (a) Representative immunofluorescent staining pictures detecting the expression of Piezo2 channel in AF tissue of the Ctrl group and IVDD group (scale bar = 150 μm). Piezo2 appeared green and nuclei were counterstained with Hoechst 33358 (blue). (b) Representative immunofluorescent staining pictures detecting the expression of Piezo2 channel in AFCs of the Ctrl group and CMS group (scale bar = 50 μm). Piezo2 appeared green and nuclei were counterstained with Hoechst 33358 (blue). Figure S2. Verification of Piezo1 knockdown by Lv-Piezo1 (n = 3). Verification of Calpain1 and Calpain2 knockdown by si-CAPN1 and siCAPN2 (n = 3). Figure S3. Statistic data of western blotting. (a-d) Western blotting analysis showing the Calpain1, Calpain2, Bax, Cleaved-Caspase3 expression in the Ctrl group, CMS group, CMS + Lv-Ctrl group, and CMS + Lv-Piezo1 group (n = 3). (e-f) Western blotting analysis showing the Bax, Cleaved-Caspase3 expression in the CMS + Yoda1 group, CMS + Yoda1 + si-Ctrl group, CMS + Yoda1 + si-CAPN1 group, and CMS + Yoda1 + si-CAPN2 group (n = 3). **P<0.01, ***P<0.001, ****P<0.0001. Table S1. Histological grading scale system. [file 13075_2023_3093_MOESM1_ESM.zip › Figure S1.tif]

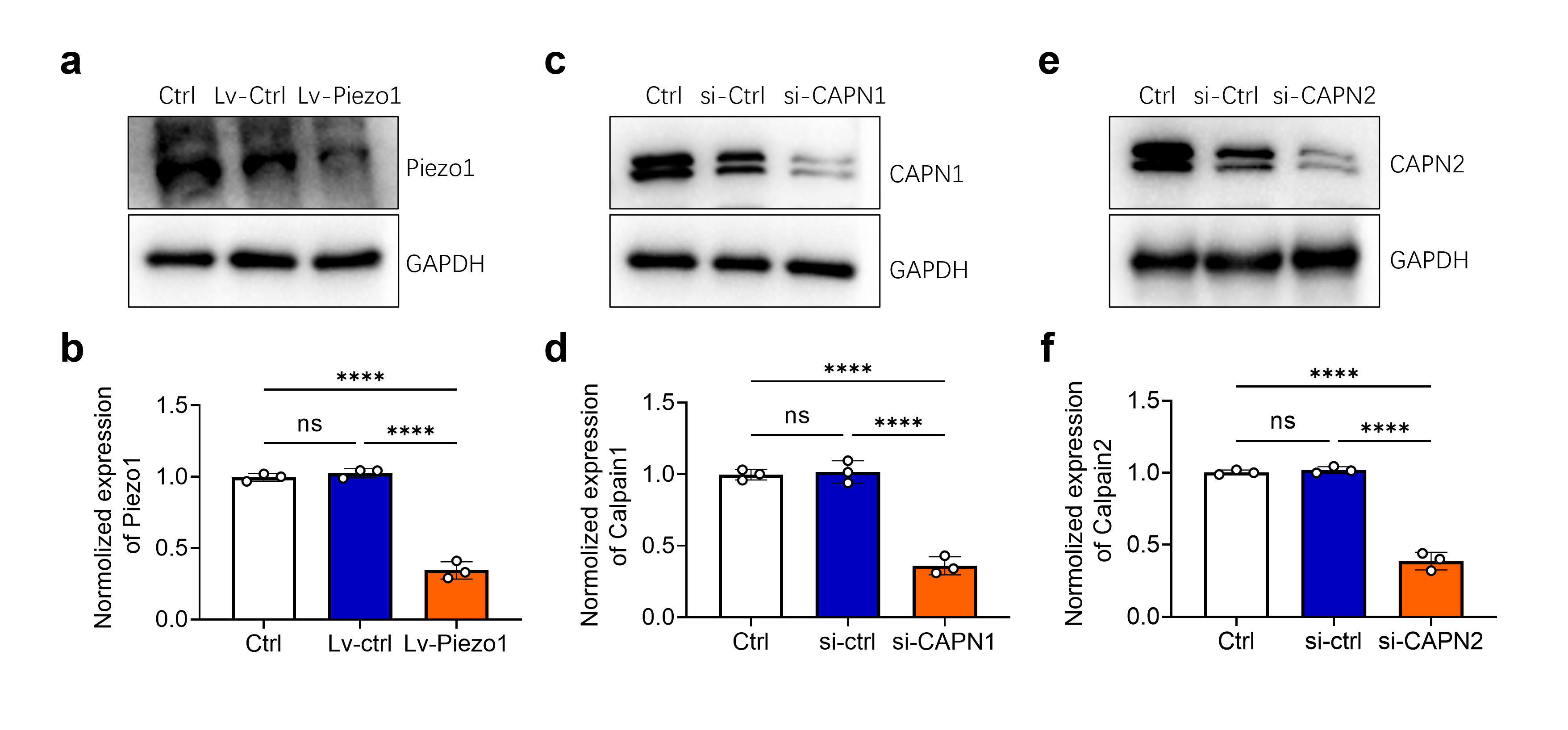

Supplement: Supplementary file 1 — Additional file 1: Figure S1. The expression of Piezo2 in AF tissue and AFCs. (a) Representative immunofluorescent staining pictures detecting the expression of Piezo2 channel in AF tissue of the Ctrl group and IVDD group (scale bar = 150 μm). Piezo2 appeared green and nuclei were counterstained with Hoechst 33358 (blue). (b) Representative immunofluorescent staining pictures detecting the expression of Piezo2 channel in AFCs of the Ctrl group and CMS group (scale bar = 50 μm). Piezo2 appeared green and nuclei were counterstained with Hoechst 33358 (blue). Figure S2. Verification of Piezo1 knockdown by Lv-Piezo1 (n = 3). Verification of Calpain1 and Calpain2 knockdown by si-CAPN1 and siCAPN2 (n = 3). Figure S3. Statistic data of western blotting. (a-d) Western blotting analysis showing the Calpain1, Calpain2, Bax, Cleaved-Caspase3 expression in the Ctrl group, CMS group, CMS + Lv-Ctrl group, and CMS + Lv-Piezo1 group (n = 3). (e-f) Western blotting analysis showing the Bax, Cleaved-Caspase3 expression in the CMS + Yoda1 group, CMS + Yoda1 + si-Ctrl group, CMS + Yoda1 + si-CAPN1 group, and CMS + Yoda1 + si-CAPN2 group (n = 3). **P<0.01, ***P<0.001, ****P<0.0001. Table S1. Histological grading scale system. [file 13075_2023_3093_MOESM1_ESM.zip › Figure S2.tif]

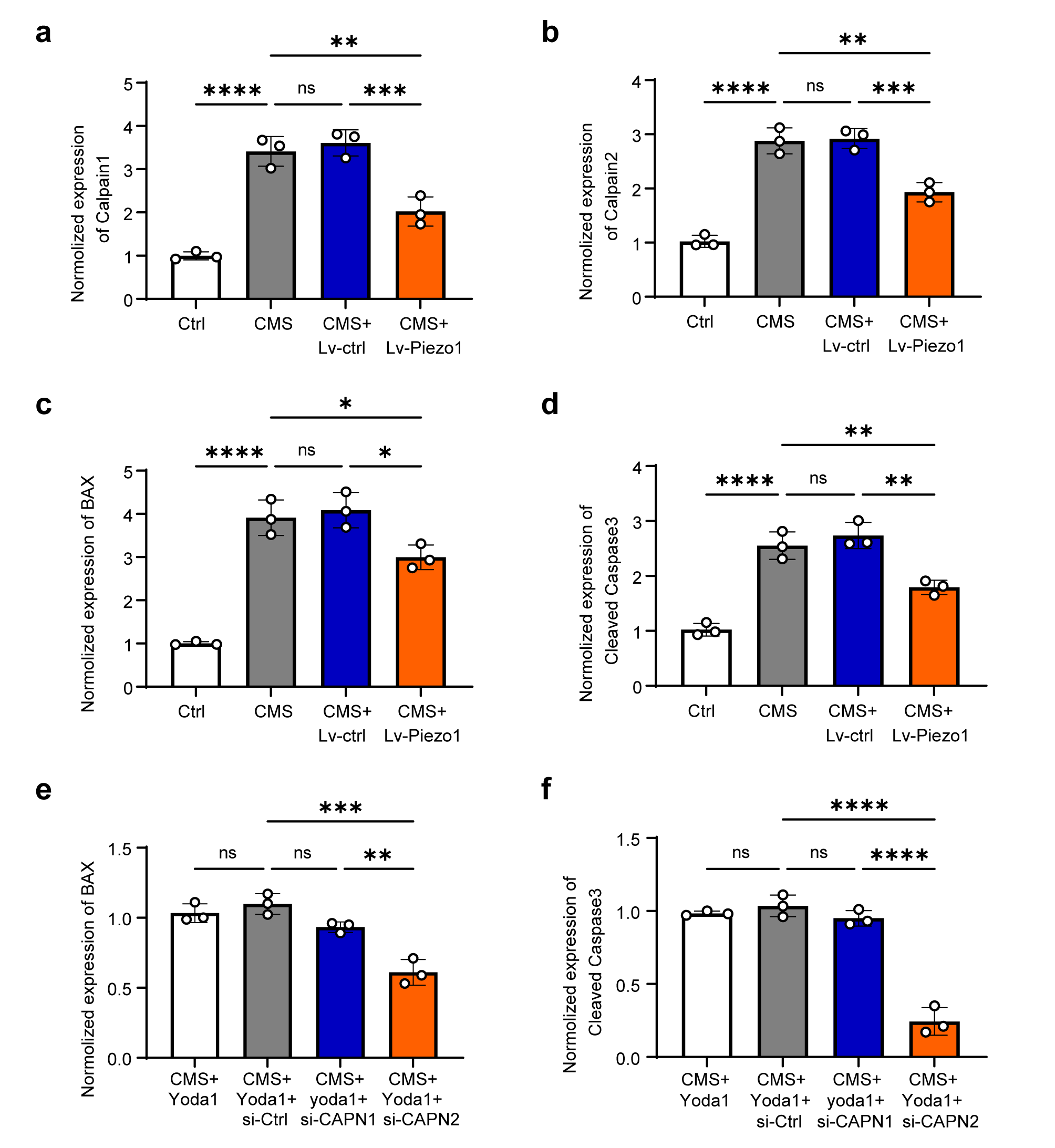

Supplement: Supplementary file 1 — Additional file 1: Figure S1. The expression of Piezo2 in AF tissue and AFCs. (a) Representative immunofluorescent staining pictures detecting the expression of Piezo2 channel in AF tissue of the Ctrl group and IVDD group (scale bar = 150 μm). Piezo2 appeared green and nuclei were counterstained with Hoechst 33358 (blue). (b) Representative immunofluorescent staining pictures detecting the expression of Piezo2 channel in AFCs of the Ctrl group and CMS group (scale bar = 50 μm). Piezo2 appeared green and nuclei were counterstained with Hoechst 33358 (blue). Figure S2. Verification of Piezo1 knockdown by Lv-Piezo1 (n = 3). Verification of Calpain1 and Calpain2 knockdown by si-CAPN1 and siCAPN2 (n = 3). Figure S3. Statistic data of western blotting. (a-d) Western blotting analysis showing the Calpain1, Calpain2, Bax, Cleaved-Caspase3 expression in the Ctrl group, CMS group, CMS + Lv-Ctrl group, and CMS + Lv-Piezo1 group (n = 3). (e-f) Western blotting analysis showing the Bax, Cleaved-Caspase3 expression in the CMS + Yoda1 group, CMS + Yoda1 + si-Ctrl group, CMS + Yoda1 + si-CAPN1 group, and CMS + Yoda1 + si-CAPN2 group (n = 3). **P<0.01, ***P<0.001, ****P<0.0001. Table S1. Histological grading scale system. [file 13075_2023_3093_MOESM1_ESM.zip › Figure S3.tif]

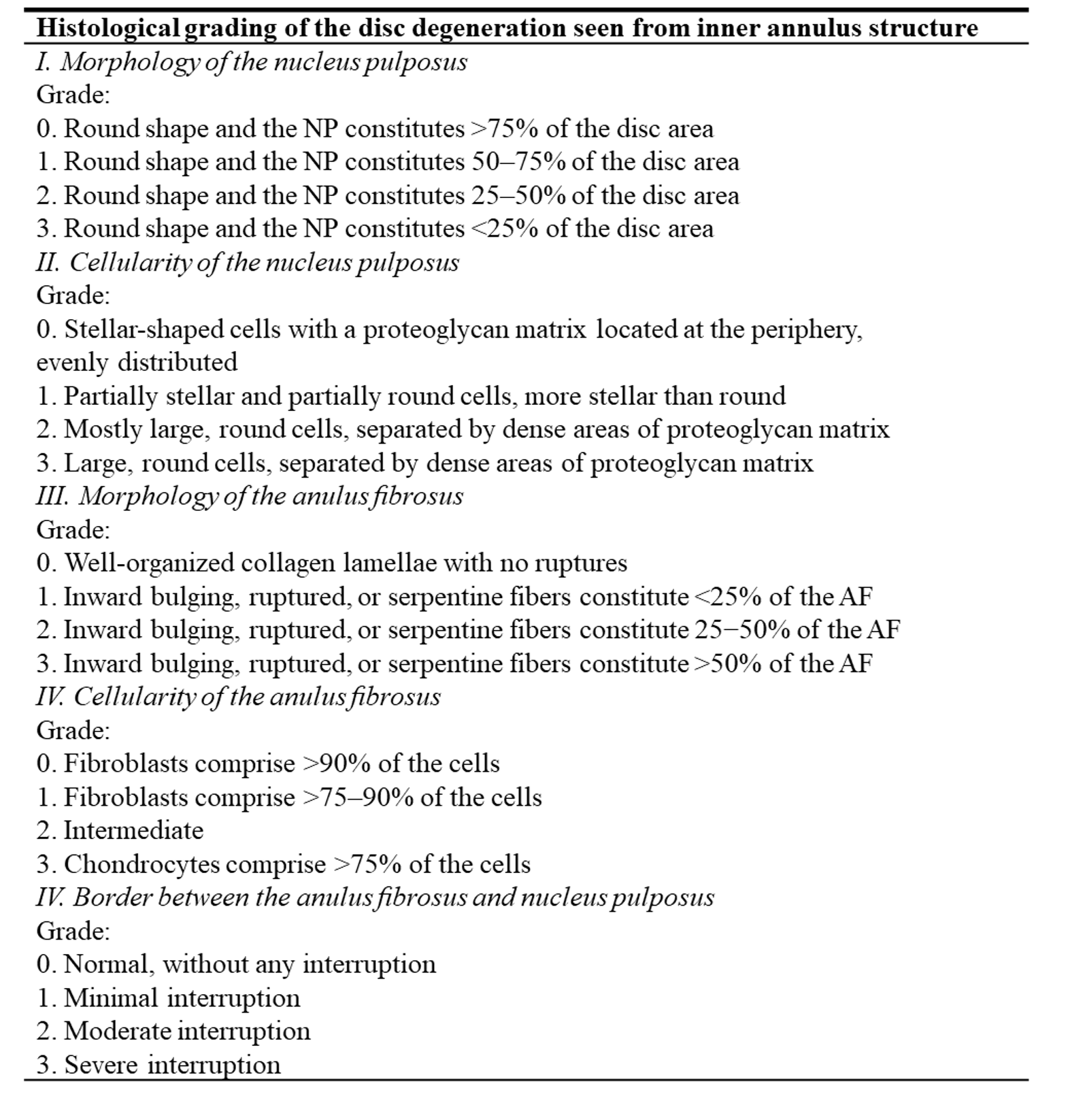

Supplement: Supplementary file 1 — Additional file 1: Figure S1. The expression of Piezo2 in AF tissue and AFCs. (a) Representative immunofluorescent staining pictures detecting the expression of Piezo2 channel in AF tissue of the Ctrl group and IVDD group (scale bar = 150 μm). Piezo2 appeared green and nuclei were counterstained with Hoechst 33358 (blue). (b) Representative immunofluorescent staining pictures detecting the expression of Piezo2 channel in AFCs of the Ctrl group and CMS group (scale bar = 50 μm). Piezo2 appeared green and nuclei were counterstained with Hoechst 33358 (blue). Figure S2. Verification of Piezo1 knockdown by Lv-Piezo1 (n = 3). Verification of Calpain1 and Calpain2 knockdown by si-CAPN1 and siCAPN2 (n = 3). Figure S3. Statistic data of western blotting. (a-d) Western blotting analysis showing the Calpain1, Calpain2, Bax, Cleaved-Caspase3 expression in the Ctrl group, CMS group, CMS + Lv-Ctrl group, and CMS + Lv-Piezo1 group (n = 3). (e-f) Western blotting analysis showing the Bax, Cleaved-Caspase3 expression in the CMS + Yoda1 group, CMS + Yoda1 + si-Ctrl group, CMS + Yoda1 + si-CAPN1 group, and CMS + Yoda1 + si-CAPN2 group (n = 3). **P<0.01, ***P<0.001, ****P<0.0001. Table S1. Histological grading scale system. [file 13075_2023_3093_MOESM1_ESM.zip › Table S1.tif]
